# Muscle and liver-specific alterations in lipid and acylcarnitine metabolism after a single bout of exercise in mice

**DOI:** 10.1038/srep22218

**Published:** 2016-02-26

**Authors:** Miriam Hoene, Jia Li, Yanjie Li, Heike Runge, Xinjie Zhao, Hans-Ulrich Häring, Rainer Lehmann, Guowang Xu, Cora Weigert

**Affiliations:** 1 Division of Clinical Chemistry and Pathobiochemistry, Department of Diagnostic Laboratory Medicine, University Hospital Tuebingen, Tuebingen, Germany; 2 Key Laboratory of Separation Science for Analytical Chemistry, Dalian Institute of Chemical Physics, Chinese Academy of Sciences, Dalian, China; 3 Department of Molecular Diabetology, Institute for Diabetes Research and Metabolic Diseases of the Helmholtz Centre Munich at the University of Tuebingen, Tuebingen, Germany; 4 German Center for Diabetes Research (DZD), Tuebingen, Germany

## Abstract

Intracellular lipid pools are highly dynamic and tissue-specific. Physical exercise is a strong physiologic modulator of lipid metabolism, but most studies focus on changes induced by long-term training. To assess the acute effects of endurance exercise, mice were subjected to one hour of treadmill running, and ^13^C_16_-palmitate was applied to trace fatty acid incorporation in soleus and gastrocnemius muscle and liver. The amounts of carnitine, FFA, lysophospholipids and diacylglycerol and the post-exercise increase in acetylcarnitine were pronouncedly higher in soleus than in gastrocnemius. In the liver, exercise increased the content of lysophospholipids, plasmalogens and carnitine as well as transcript levels of the carnitine transporter. ^13^C_16_-palmitate was detectable in several lipid and acylcarnitine species, with pronounced levels of tracer-derived palmitoylcarnitine in both muscles and a strikingly high incorporation into triacylglycerol and phosphatidylcholine in the liver. These data illustrate the high lipid storing activity of the liver immediately after exercise whereas in muscle, fatty acids are directed towards oxidation. The observed muscle-specific differences accentuate the need for single-muscle analyses as well as careful consideration of the particular muscle employed when studying lipid metabolism in mice. In addition, our results reveal that lysophospholipids and plasmalogens, potential lipid signalling molecules, are acutely regulated by physical exercise.

Certain lipids such as ceramides, diacylglycerols, and acylcarnitines have been shown to activate intracellular signalling cascades and to regulate metabolic pathways^1^,^2^,^3^,^4^. Intracellular accumulation of these lipids in liver and skeletal muscle is frequently related to a chronic inflammatory state and to the development of metabolic diseases^5^,^6^. However, lipid bioactivity *per se* is a physiological event and it is only the deregulated production and storage of lipids that leads to a non-physiological activation of signalling pathways^7^. Physical exercise induces tissue-specific alterations in substrate supply and utilization that are evident as increased turnover of fatty acids^8^ and elevated levels of acylcarnitines^9^. Previous studies addressed the hepatic lipid response to long-term training^10^ or the exercise-dependent regulation of the intramyocellular triglyceride pools in skeletal muscle^11^, but the acute regulation of other lipid species by one bout of exercise is less well understood. Thus, changes in the levels of individual lipid signaling molecules could play a role in the acute response to physiological challenges that affect lipid metabolism.

While it is reasonable to expect differences in the regulation of lipid species between organs such as liver and muscle, less attention is usually paid to the distinct metabolism of individual skeletal muscles. Muscles contain different myofibres with specific metabolic and contractile properties. The main classification for different types of muscle fibres is into “slow” or “fast”, based on their velocity of shortening. Fast fibres are most powerful in maximal movements, while slow fibres are most efficient during slow locomotion^12^. Fast fibres rely more on glucose for energy production than slow fibres which have a higher content of mitochondria and thus a higher capacity for fatty acid oxidation. The two calf muscles gastrocnemius and soleus differ in their fibre composition, particularly in mice, the model system most frequently used for comparative metabolic studies of different tissues^13^,^14^. Since both are plantar flexors, these two muscles are particularly suited for being compared in an exercise setting.

To study the acute regulation of lipid species in skeletal muscles and liver by endurance exercise, untrained mice were subjected to one hour of moderately intense treadmill running. This protocol has previously been shown to provoke metabolic and transcriptional responses in both tissues^15^,^16^. Moreover, we applied ^13^C_16_-palmitate immediately after the exercise bout or in control mice, a method that has successfully been applied to trace fatty acid incorporation into lipids in cultured cells^17^ and in humans^18^,^19^. Free fatty acids (FFA), acylcarnitines and lipids were assessed by ultra-high performance liquid chromatography-mass spectrometry (UHPLC-MS).

## Results

### The lipid composition of the glycolytic gastrocnemius muscle is different from the oxidative soleus

**Figure 1:**
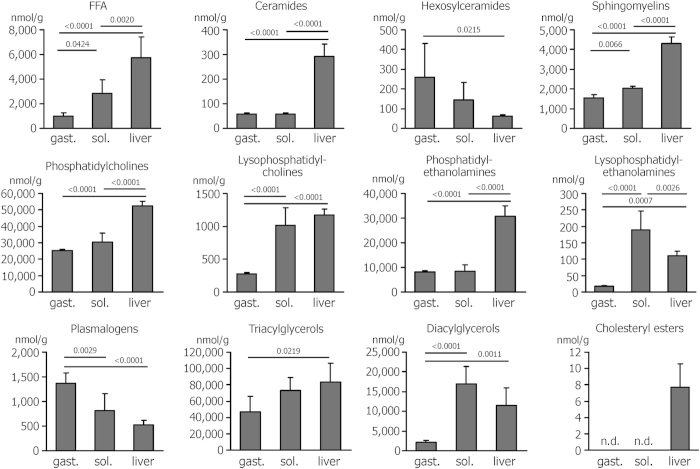
Lipid composition of gastrocnemius (gast.) and soleus (sol.) muscles and liver of sedentary mice. 33 free fatty acid (FFA) and 199 lipid species were detected by UHPLC-MS and summed up according to lipid class. Cholesteryl esters were only detected in the liver. Values are means ± SD from n = 4–6 mice. Concentrations of individual FFA and lipids are provided in the Supplementary Tables S-1 and S-2.

We detected a total of 33 FFA and 199 lipid species in gastrocnemius and soleus muscles and in the liver of sedentary C57Bl/6NCrl mice (see Supplementary Tables S-1 and S-2 for the complete results). Pronounced differences were visible between liver and muscle, but also between the two muscles. The liver was characterized by a higher content of FFA, ceramides, sphingomyelins and phospholipids and by detectable amounts of cholesteryl esters (Fig. 1). Of the two muscles, the soleus exhibited a lipid pattern that was more similar to the liver, with higher levels of FFA, lysophosphatidylcholines, lysophosphatidylethanolamines, and diacylglycerols than the gastrocnemius, while plasmalogens were lower (Fig. 1). In addition, polyunsaturated fatty acids accounted for 10 ± 2% of all FFA in the gastrocnemius and 16% ± 4% and 18% ± 2%, respectively, in soleus and liver (Supplementary Table S-1). Similarly, monounsaturated fatty acids made up for 20% ± 3% in gastrocnemius, 26% ± 6% in soleus, and 30 ± 5% in the liver (Supplementary Table S-1).

### Exercise causes an acute increase in hepatic lysophospholipid and plasmalogen species

**Figure 2:**
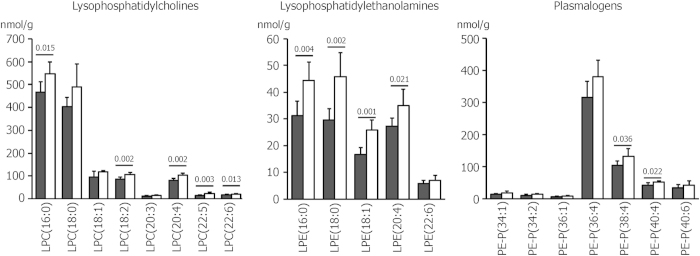
Amount of lysophosphatidylcholines, lysophosphatidylethanolamines and plasmalogens (PE-P) in livers of control mice (black bars) and after an acute bout of treadmill exercise (white bars). Values are means ± SD of n = 6 mice.

While most lipids were not significantly altered in either muscle or in the liver upon an acute bout of exercise, we did observe an increase in total hepatic lysophospholipid and plasmalogen levels (Supplementary Table S-3). The increase was significant for most lysophosphatidylcholine and lysophosphatidylethanolamine species (Fig. 2). The latter also showed a particularly high increase of, in sum, 43% (Supplementary Table S-3). Since the summed phosphatidylcholine and phosphatidylethanolamine levels were not increased upon exercise (Supplementary Table S-3), the lysophospholipid to phospholipid ratios were also elevated (LPC/PC 2.3% ± 0.2% before vs. 2.7% ± 0.3% after exercise; LPE/PE 0.36% ± 0.01% before vs. 0.48% ± 0.11% after exercise). Among the seven plasmalogens detected, two species, PE-P(38:4) and PE-P(40:4), exhibited a significant exercise-induced increase (Fig. 2).

### Carnitine and acylcarnitine contents are tissue-specific and acutely affected by exercise

**Figure 3:**
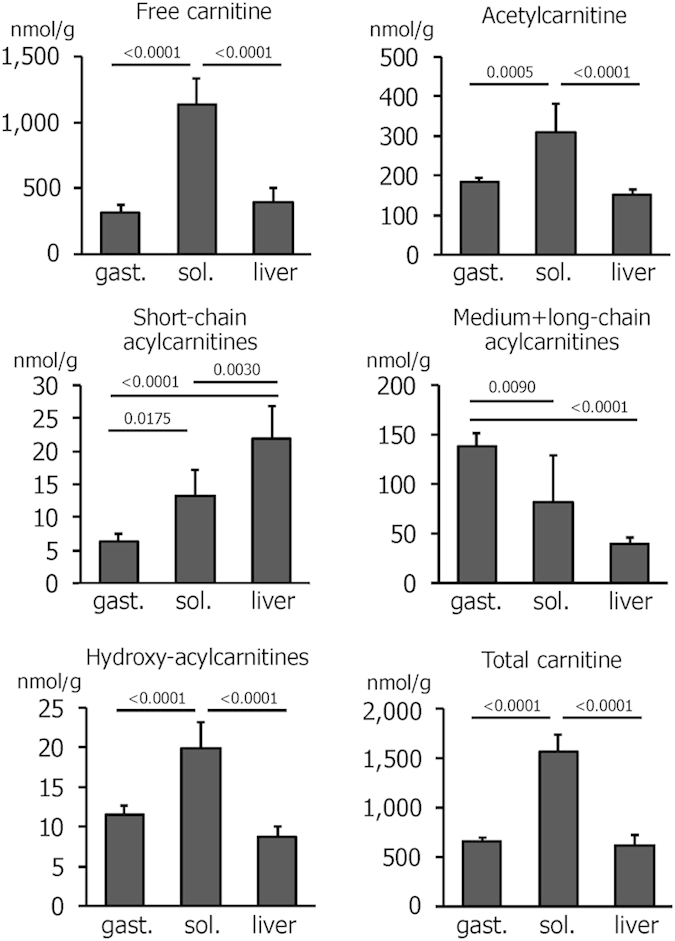
Acylcarnitine content of gastrocnemius (gast.) and soleus (sol.) muscles and liver of sedentary mice. In addition to free and acetylcarnitine, 39 acylcarnitine species were detected by UHPLC-MS and summed up according to chain length or hydroxylation. Values are means ± SD from n = 6 mice. The values for the individual carnitine species can be found in the Supplementary Table S-4.

By targeted UHPLC-MS analysis, we detected free carnitine, acetylcarnitine and 39 acylcarnitines with chain lengths from 3–20 carbon units (for the complete results see Supplementary Table S-4). The most notable difference between the two muscles could be observed for free carnitine, which was more than 3.5 times higher in the soleus (Fig. 3), and propionylcarnitine, which was 4 times higher (Supplementary Table S-4). Similarly, acetylcarnitine and short-chain and hydroxylated acylcarnitines (Fig. 3) were also significantly higher in the soleus. Accordingly, the total carnitine content was more than twice as high in the soleus than in the gastrocnemius (Fig. 3). In contrast, medium- and long-chain acylcarnitine levels were significantly lower in the soleus (Fig. 3). The total and free carnitine content of the liver was more similar to the gastrocnemius than to the soleus (Fig. 3). The sum of hepatic short-chain acylcarnitines, however, was strikingly high (Fig. 3) due to a relatively high amount of propionylcarnitine in the liver, 12.8 ± 4.1 nmol/g compared to 6.8 ± 2.6 nmol/g in the soleus and 1.7 ± 0.4 in the gastrocnemius (Supplementary Table S-4).

In both muscles, the levels of acetylcarnitine, short-chain and hydroxy-acylcarnitines were significantly increased by exercise (Table 1). The increase in acetylcarnitine above sedentary levels was more pronounced in the soleus, 61% compared to only 15% in the gastrocnemius. Similarly, the increase in hydroxy-acylcarnitines was more pronounced in the soleus. The amounts of free and total carnitine were not altered in either muscle (Table 1).

### Exercise causes an acute increase in hepatic carnitine

**Figure 4:**
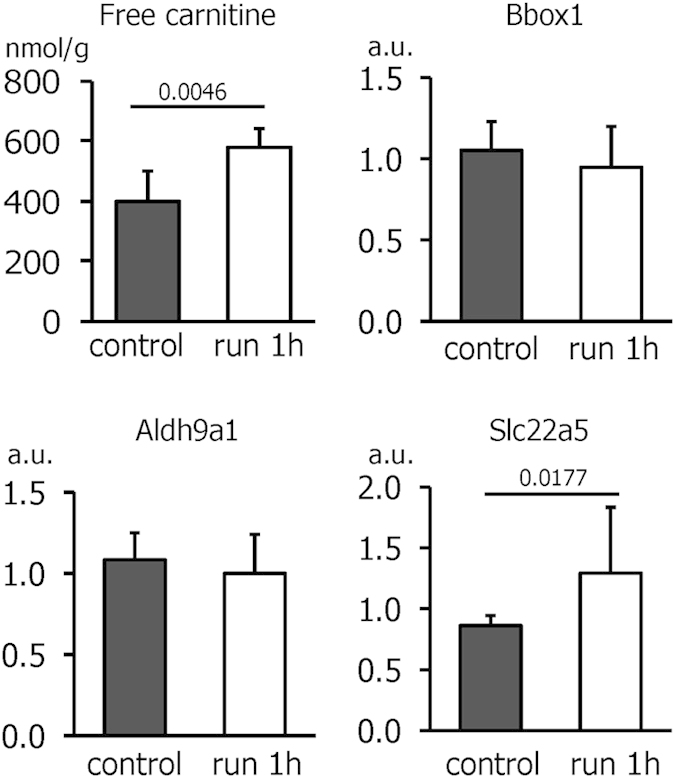
Amount of free carnitine and of Bbox1, Aldh9a1, and Slc22a5 mRNA in livers of control mice or after an acute bout of treadmill running. Values are means ± SD (n = 6 for carnitine, n = 10–12 for mRNAs); a.u., arbitrary units.

Interestingly, treadmill running acutely elevated the level of free carnitine in the liver (Fig. 4), resulting in an increase in total carnitine (Supplementary Table S-3). Acetylcarnitine levels were decreased, while other acylcarnitines were not affected by exercise in the liver (Supplementary Table S-3). Data from a whole genome expression analysis of mouse liver after an acute bout of exercise^20^ pointed towards an upregulation of the carnitine transporter solute carrier family (Slc)22a5 which encodes for the organic cation transporter 2 (OCTN2). To investigate whether the increase in hepatic carnitine could be related to a higher carnitine uptake or to an elevated synthesis, we determined the expression of Slc22a5 and of the biosynthetic enzymes gamma-butyrobetaine hydroxylase (Bbox)1 and aldehyde dehydrogenase (Aldh)9a1 (Fig. 4). While Bbox1 and Aldh9a1 were unaltered after exercise, we observed a significant increase in the mRNA content of the carnitine transporter Slc22a5 (Fig. 4).

### The acute palmitate flux differs between liver and muscle

**Figure 5:**
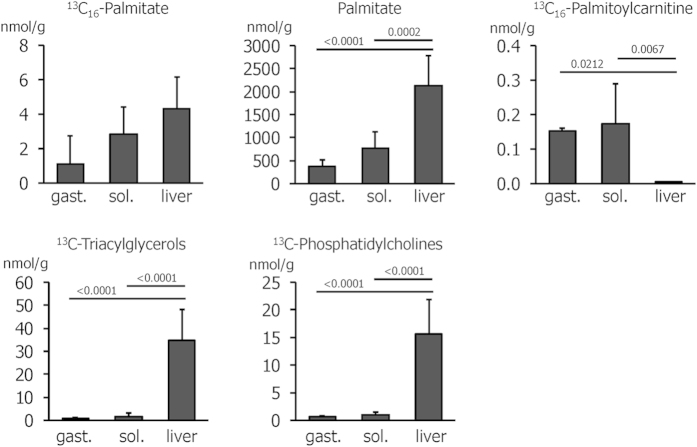
Amounts of ^13^C-labelled palmitate, palmitoylcarnitine, triacylglycerols and phosphatidylcholines and of endogenous palmitate in gastrocnemius (gast.) and soleus (sol.) muscles and liver of sedentary mice. Values are means of n = 4–6 mice.

General lipid and acylcarnitine patterns revealed tissue- and metabolic state-dependent differences between soleus and gastrocnemius muscles and liver. To additionally track the acute flux of FFA, we injected a small amount of ^13^C_16_-labelled palmitate as a tracer. Free labelled palmitate was detectable in the tissues ten minutes after the intravenous injection, with the highest amount in the liver, followed by soleus and gastrocnemius (Fig. 5). Overall, the pattern of labelled palmitate resembled that of endogenous palmitate (Fig. 5). In addition to the free tracer, several labelled acylcarnitine and lipid species were detectable (Table 2). Labelled palmitoylcarnitine (Fig. 5) was predominantly found in the muscles. We additionally detected labelled tetradecanoylcarnitine in the soleus and lauroyl- and butyryl-carnitine in all tissues (Table 2).

The liver showed the highest amount of palmitate incorporation into lipids: The sum of labelled triacylglycerols in sedentary mice amounted to 34.6 ± 13.4 nmol/g in the liver, 1.7 ± 1.5 nmol/g in the soleus and 0.8 ± 0.3 nmol/g in the gastrocnemius (Fig. 5), and 15.7 ± 6.2 nmol/g labelled phosphatidylcholines were detected in the liver compared to less than 1 nmol/g in soleus and gastrocnemius (Fig. 5). In addition, palmitate was incorporated into individual phosphatidylcholine and triacylglycerol species in a tissue-specific manner: PC(32:0) was the only labelled phosphatidylcholine in the muscles, but in the liver, it accounted for less than 5% of the labelled phosphatidylcholines (Table 2). Here, PC(34:2) and PC(34:1) were most abundant. Similarly, TG(50:1) was the highest labelled triacylglycerol species in both muscles, while TG(52:3) dominated in the liver. Interestingly, these differences were only apparent for labelled, but not for unlabelled lipids: Endogenous TG(52:3) was the most abundant lipid in all tissues, accounting for 16 ± 1% of all lipids in the liver and 15 ± 1% in either muscle, and PC(34:2) was the highest or second highest phosphatidylcholine species in all three tissues (Supplementary Table S-2). Compared to the corresponding unlabelled lipid, 0.1% of TG(50:1) was labelled in the liver and less than 0.05% were labelled in both muscles. Labelled PC(32:0) accounted for approximately 0.1% of total PC(32:0) in all three tissues. With the exception of lower ^13^C_16_-PC(32:0) in the gastrocnemius, the incorporation of labelled palmitate into acylcarnitines and triacylglycerols was not detectably altered by acute exercise (Table 2).

## Discussion

The aim of our study was to identify individual lipids and lipid metabolites that are acutely regulated by physical exercise and to compare the lipid composition and dynamics of muscle and liver. We also focused on differences between the soleus muscle, which is composed of more slow, oxidative fibers, and the gastrocnemius with predominantly fast-contracting fibres.

Mice are valuable animal models to study the protective mechanisms by which physical exercise affects the lipid dynamics of different tissues. However, little is known about the differences in lipid composition and dynamics of individual mouse muscles. Given its ten times higher content of type I fibres^14^, we expected the soleus to contain more lipids and intermediates of fatty acid metabolism. This difference was most pronounced for free carnitine, but also obvious for total carnitine and FFA. The soleus has a higher rate of fatty acid oxidation^21^ and a greater capacity for carnitine uptake than the gastrocnemius, due to a higher content of the carnitine transporter Slc22a5 (OCTN2)^22^. The fatty acid oxidation product acetylcarnitine was not only higher in the soleus under basal conditions, but also showed a greater increase after an acute bout of treadmill exercise. This is in line with a recent study that showed acetylcarnitine enrichment in individual muscle fibers following *in situ* contraction^23^. The acetylcarnitine pool acts as a buffering system with two functions, to store acetyl groups and to prevent CoA from being trapped as acetyl-CoA^24^. In addition, the resulting decrease in the ratio of acetyl-CoA to CoA can promote pyruvate dehydrogenase activity^25^. The same pattern of higher basal levels and a greater induction by exercise could not only be observed for the short-chain acylcarnitines, but also for hydroxy-acylcarnitines, suggesting that the higher rate of oxidation went along with a higher accumulation of incomplete oxidation intermediates in the soleus.

The muscle content of medium- and long-chain acylcarnitines was unchanged upon exercise, in contrast to a previous study^26^. Since the animals were exercised till exhaustion in the latter study, it is conceivable that higher intensities are required for an accumulation inside skeletal muscle. Moreover, the mice were immobilized by anaesthesia in the present study to allow for the intravenous application of the tracer. This could have reduced the acute effects of exercise on acylcarnitine levels in skeletal muscle. We chose immobilization by anaesthesia since it causes less acute stress and pain than restraint and tail vein injection of non-anaesthetized mice^27^. Notably, the differences in acylcarnitine levels between red and white muscle have been shown to be conserved in anaesthetized mice^28^.

When comparing the summed levels of the different lipid classes, the soleus showed some similarities to the liver, including higher concentrations of FFA, lysophospholipids, diacylglycerols, and lower plasmalogens, while other phospholipids were not significantly different between the two muscles. This similarity was also reflected by higher percentages of mono- and polyunsaturated fatty acids in liver and soleus.

The liver was characterized by a generally higher content of membrane lipids, by particularly high levels of ceramides and phosphatidylethanolamines, and lower levels of plasmalogens. Cholesteryl esters were only present in the liver. Upon exercise, we observed an intriguing increase of lysophosphatidylcholines, lysophosphatidylethanolamines and plasmalogens in the liver. The bulk of plasmalogens synthesized by the liver are secreted^29^. Thus, their accumulation during and after exercise might be related to an acute reduction of lipoprotein secretion^30^. Increased lysophosphatidylcholine concentrations in plasma have previously been associated with insulin sensitivity and a metabolically benign fatty liver^31^,^32^ while inducing non-alcoholic fatty acid liver in mice increases hepatic lysophosphatidylcholine acyltransferase expression, thereby reducing serum lysophosphatidylcholine levels^33^. Less is known about the regulation of intrahepatic lysophospholipids by acute metabolic challenges. The increase in the lysophospholipid to phospholipid ratios after exercise indicates an activation of intrahepatic phospholipases. Phospholipase A2 can be activated by cytokines and PPARα-dependent signalling^34^,^35^, both of which could play a role in the liver during physical exercise^36^,^37^. Understanding the biological function of intracellular lysophospholipids has just started, but recent data suggest, in turn, a role in the regulation of PPARα target genes^34^. Further studies are needed to evaluate the potential relevance of hepatic lysophospholipid production for the adaptation of the liver to physical exercise.

Both free and total carnitine concentrations were elevated in the liver upon exercise. This was likely related to an increased uptake since the mRNA of the carnitine transporter Slc22a5/OCTN2 was acutely upregulated. Previously, regular training has been shown to reverse a high-fat diet-induced reduction in hepatic carnitine concentrations by upregulating carnitine transporter and biosynthetic enzymes in mice^38^. Our results indicate that at least acutely, the increase in hepatic carnitine is caused by physical exercise *per se* and does not depend on secondary effects of training such as weight loss.

Besides a mere quantification, we also tracked acute lipid fluxes by an intraperitoneal application of ^13^C_16_-labelled palmitate, a method that has successfully been applied in cell culture^17^ and human studies^18^,^19^. Our study showed that stable isotope tracers of fatty acids can be employed to study lipid metabolism *in vivo* in mice and revealed several tissue-specific differences: Labelled palmitoylcarnitine was present in all tissues analyzed; however, its hepatic levels amounted to only 0.004 nmol/g in control mice despite a relatively high ^13^C-palmitate concentration of 4 nmol/g. At the same time, several hepatic triacylglycerol and phosphatidylcholine species were labelled to a high degree, amounting to a total of 50 nmol/g. This was in contrast to the muscles where lipids were labelled to a low degree whereas the amount of ^13^C_16_-palmitoylcarnitine was strikingly high. Thus, our results employing ^13^C_16_-palmitate extend our previous findings^39^ by showing that excess amounts of plasma FFA in the recovery phase following an acute bout of endurance exercise are expeditiously incorporated not only into triacylglycerols, but also into phospholipids in the liver. With the exception of one phosphatidylcholine species in the gastrocnemius, we did not detect significant differences in ^13^C_16_-labelling after exercise, despite our explorative approach which did not correct for multiple testing. However, we cannot exclude that the number of mice studied was too small to detect more subtle differences in lipid utilization.

Notably, the overall hepatic phospholipid content does not increase at the same time as triacylglycerols during post-exercise recovery^39^ or after fasting^40^, another state of transiently elevated FFA. Our results suggest the fatty acid composition of both phosphatidylcholines and triacylglycerols to be in a constant state of flux, and this flux could differ between liver and muscle: In the liver, TG(52:3) was the quantitatively most abundant endogenous triacylglycerol species and this species also exhibited the highest degree of palmitate labelling. TG(52:3) was also the most abundant endogenous triacylglycerol species in both muscles. Unexpectedly, however, another species, TG(50:1) exhibited the highest labelling degree in the muscles. Moreover, the palmitate tracer was preferentially incorporated into a desaturated phosphatidylcholine species in the liver and into a saturated species in both muscles. One mechanism underlying these organ-specific differences in lipid handling could be the greater content of FFA and particularly of mono-unsaturated FFA in the liver; however, FFA and the percentage of mono-unsaturated FFA were also higher in the soleus compared to the gastrocnemius, pointing towards additional regulatory differences. While ^13^C_16_-palmitate was the only labelled fatty acid detected, it is still conceivable that some of the tracer incorporated into lipids was desaturated, and this process could have occurred at a higher rate in the liver.

To summarize, we could show that endurance exercise acutely increases the concentrations of lysophospholipids, plasmalogens, and carnitine in the liver. By tracing fatty acid metabolism with stable isotope-labelled palmitate, we could further show that excess fatty acids are disposed of differently in skeletal muscles and in the liver, where storage in the form of triacylglycerol prevails. An additional finding of our study is that in mice, the two synergistic muscles gastrocnemius and soleus differ greatly in their lipid and carnitine profiles and in the acylcarnitine response to an acute bout of endurance exercise. This is particularly relevant given that the soleus is the mouse muscle with the highest content of slow, oxidative fibres and therefore most similar to human skeletal muscle. Different conclusions might be drawn depending on which muscle is studied when aiming to elucidate the impact of a physiologic or pathophysiologic challenge on metabolic pathways.

## Experimental Procedures

### Chemicals and internal standards

Liquid chromatography grade solvents were purchased from Merck (Darmstadt, Germany) or Tedia (Fairfield, OH, USA). Ammonium formate of analytical grade and ^13^C_16_-palmitate were obtained from Sigma-Aldrich (St.Louis, MO, USA). The internal standards d_3_-carnitine, d_3_-acetylcarnitine, d_3_-propionylcarnitine, d_3_-decanoylcarnitine, d_3_-palmitoylcarnitine and d_4_-docosanoic acid were purchased from Ten Brink (Amsterdam, The Netherlands). d_4_-palmitic acid was from Cambridge Isotope Laboratories (Tewksbury, MA, USA). Ceramide (CER) (d18:1/17:0), diacylglycerol (DAG) (14:0/14:0), lysophosphatidylcholines (LPC) (15:0) and (19:0), phosphatidylcholines (PC) (17:0/17:0) and (19:0/19:0), phosphatidylethanolamine (PE) (17:0/17:0), sphingomyelin (SM) (d18:1/12:0) and triacylglycerols (TAG) (17:0/17:0/17:0) and (15:0/ 15:0/15:0) were purchased from Avanti Polar Lipids, Inc. (Alabaster, Alabama, USA).

The palmitate solution for injection was prepared as follows: Firstly, ^13^C_16_-palmitate from a 200 mM ethanol stock was added to a concentration of 6 mM to a 10% BSA solution (Fraction V, very low endotoxin, fatty acid free; Serva, Heidelberg, Germany) in sterile 0.9% NaCl. BSA-coupling of palmitate was achieved by shaking incubation over night at 37 °C. Then, the solution was further diluted with isotonic saline to a final palmitate concentration of 4.2 mM.

### Animal care, exercise protocol and tracer application

All animal experiments were conducted in accordance with the national guidelines of laboratory animal care and approved by the local governmental commission for animal research (Regierungspraesidium Tuebingen, Baden-Wuerttemberg, Germany). Male C57Bl/6NCrl mice were purchased from Charles River (Sulzfeld, Germany) and kept under an inverted light-dark cycle (light period 21:00-9:00) with free access to chow (C1000 without vitamin C, Altromin, Lage, Germany) and tap water. Exercise experiments were performed between 9:00 and 13:00 in 12 week-old animals (n = 6). All mice were habituated to treadmill running (Mouse Accupacer treadmill with motorized grade adjust, Hugo Sachs Elektronik, March-Hugstetten, Germany) for 10 min on three non-successive days. The last habituation took place one week before the experiment.

The acute bout of exercise consisted of 5 min of warm-up at a speed of 5 m/min, followed by 60 min running at 13 m/min and 13° uphill slope. To ensure similar feeding state and exposure, control mice were placed for 60 min in new cages without food, but access to water, in the same room. Immediately after completing the treadmill run, mice were anaesthetized with an intraperitoneal injection of ketamine and xylazine (110 mg/kg and 5 mg/kg body weight, respectively). Within 5 minutes after anaesthetic administration, mice were placed on a heating pad and injected into the lateral tail vein with 21nmol/g body weight of ^13^C_16_-palmitate. After another ten minutes, mice were killed and exsanguinated by decapitation. Liver and hindlimb muscles were quickly dissected, flash-frozen in liquid nitrogen and stored at −80 °C. For mRNA analysis, mice (n = 10–12) were similarly exercised but sacrificed immediately without prior tracer application.

### Metabolite extraction and analysis

A methyl-*tert*-butyl ether (MTBE)-based two-phase lipid extraction was performed as previously described^41^ using 4 ml of MTBE for 30 mg of freeze-dried liver or one complete gastrocnemius and 1 ml of MTBE for one soleus muscle. Acylcarnitines and FFA were extracted from freeze-dried liver or muscle using 80% acetonitrile (1 ml for 10 mg dry liver or one soleus, 3 ml for one gastrocnemius) including internal standards as follows: After homogenization in a TissueLyser (Qiagen, Hilden, Germany), samples were centrifuged for 20 min at 14,000 × g, 4 °C and the supernatant was evaporated to dryness in a speed-vac.

Acylcarnitines and FFA were quantified by UHPLC coupled online via electrospray ionization to a triple quadrupole mass spectrometer (LC/MS QQQ 6460, Agilent, Santa Clara, CA, USA) as previously described^41^. For ^13^C-labeled acylcarnitines and FFA species, the MRM transitions (precursor and product ion pairs) were set based on their ^13^C-labeling patterns. Peak areas were integrated through MassHunter Quantitative Analysis software (Agilent, Santa Clara, CA, USA) and quantified using their appropriate internal standards. Natural isotope correction was performed for quantification of ^13^C-labeled metabolites where appropriate.

A UHPLC coupled online via electrospray ionization with LTQ Orbitrap XL (Thermo Fisher Scientific) hybrid mass spectrometer was used for the lipidomics analysis as previously described^41^. Lipid assignment was performed based on high resolution mass measurement (<5 ppm) and MS/MS fragment ion information. ^13^C-labeled lipid species co-eluted in LC with their ^12^C-counterparts and showed exact m/z shifts compared to ^12^C-counterparts based on their ^13^C-labeling patterns (<5 ppm), which were used for identification of labelled lipids. Quantitative levels of lipid species were determined by high resolution EIC (extracted ion chromatogram) utilizing the Thermo Xcalibur software (V2.1, Thermo Fisher Scientific) and normalized to the corresponding internal standards. Lipid nomenclature followed the recommendations of the Lipid Maps consortium.

### RNA isolation, RT-PCR and real-time quantitative PCR analysis

Frozen tissue was homogenized in a TissueLyser (Qiagen, Hilden, Germany) and RNA was extracted with the RNeasy Fibrous Tissue Kit (Qiagen, Hilden, Germany). Reverse transcription of total RNA (1 μg) was performed in a volume of 20 μl using random hexamer primers with the First strand cDNA synthesis kit for RT-PCR (Roche, Mannheim, Germany). Aliquots were then submitted to online quantitative PCR with the Light Cycler system (Roche, Mannheim, Germany) using the QuantiFast SYBR Green PCR Kit (Qiagen, Hilden, Germany) and QuantiTect Primer Assays (Qiagen, Hilden, Germany). The mRNA content was normalized to the housekeeping transcript β-actin and is shown in arbitrary units.

### Statistical analysis

Statistical analysis of tissue-specific differences was performed by Welch ANOVA, followed by pairwise comparison using Tukey-Kramer honestly significant difference test when a significant effect of tissue was given. The effect of exercise was assessed using a two-sided Student’s t-test. Statistical comparisons were performed on the sums of lipid classes, and individual lipids were only analysed within significant lipid classes. For labelled lipids, where some classes were only represented by a single species, all individual lipids were tested. Statistical analyses were performed using JMP 11 (SAS Institute Inc., Cary, NC, USA). Values are shown as means and standard deviations (SD). A p-value < 0.05 was considered significant.

## Additional Information

**How to cite this article**: Hoene, M. *et al.* Muscle and liver-specific alterations in lipid and acylcarnitine metabolism after a single bout of exercise in mice. *Sci. Rep.* **6**, 22218; doi: 10.1038/srep22218 (2016).

## Supplementary Material

Supplementary Dataset 1

## Figures and Tables

**Table 1:** Carnitine and acylcarnitine content in muscles of sedentary or acutely exercised mice (n = 4–6).

		Control		Run 1 h		p-value	change
		Mean nmol/g	SD	Mean nmol/g	SD		
Gastrocnemius muscle	Free carnitine	319	57	307	56	n.s.	
	Acetylcarnitine	186	9	214	28	0.0400	15%
	Short-chain acylcarnitines	6	1	10	3	0.0302	57%
	Medium + long-chain acylcarnitines	139	13	122	21	n.s.	
	Hydroxy-acylcarnitines	11	1	14	2	0.0268	20%
	Total carnitine	661	42	667	39	n.s.	
Soleus muscle	Free carnitine	1137	193	1091	153	n.s.	
	Acetylcarnitine	309	73	496	71	0.0011	61%
	Short-chain acylcarnitines	13	4	19	4	0.0286	46%
	Medium + long-chain acylcarnitines	81	48	85	45	n.s.	
	Hydroxy-acylcarnitines	20	3	31	11	0.0499	54%
	Total carnitine	1561	182	1723	186	n.s.	

**Table 2:** ^13^C-labelled palmitate, acylcarnitine and lipid content of gastrocnemius and soleus muscles and liver of sedentary control mice or after one hour of moderately intense running (n = 4–6).

		Control		Run 1 h	
		Mean nmol/g	SD	Mean nmol/g	SD
Gastrocnemius FFA	^13^C_16_-Palmitate	1.0953	1.6591	0.5411	0.5050
Soleus FFA	^13^C_16_-Palmitate	2.8600	1.5426	1.2517	0.7341
Liver FFA	^13^C_16_-Palmitate	4.3313	1.8261	5.6469	1.4409
Gastrocnemius carnitines	^13^C_4_-Butyrylcarnitine (C4)	0.1082	0.0211	0.1030	0.0069
	^13^C_12_-Lauroylcarnitine (C12)	0.0138	0.0045	0.0101	0.0015
	^13^C_16_-Palmitoylcarnitine (C16)	0.1538	0.0080	0.1331	0.0406
Soleus carnitines	^13^C_4_-Butyrylcarnitine (C4)	0.1075	0.0211	0.1175	0.0409
	^13^C_12_-Lauroylcarnitine (C12)	0.0210	0.0051	0.0153	0.0072
	^13^C_14_-Tetradecanoylcarnitine (C14)	0.0259	0.0152	0.0213	0.0038
	^13^C_16_-Palmitoylcarnitine (C16)	0.1750	0.1150	0.1672	0.0652
Liver Carnitines	^13^C_4_-Butyrylcarnitine (C4)	0.0977	0.0020	0.1034	0.0089
	^13^C_12_-Lauroylcarnitine (C12)	0.0069	0.0016	0.0076	0.0024
	^13^C_16_-Palmitoylcarnitine (C16)	0.0044	0.0003	0.0081	0.0076
Gastrocnemius lipids	^13^C_16_-Phosphatidylcholine (32:0)*	0.6808	0.1164	0.4706	0.1296
	^13^C_16_-Triacylglycerol (48:0)	0.1893	0.0681	0.1416	0.0784
	^13^C_16_-Triacylglycerol (50:1)	0.4726	0.1344	0.4248	0.1239
	^13^C_16_-Triacylglycerol (52:1)	0.0689	0.0074	0.0502	0.0285
	^13^C_16_-Triacylglycerol (52:2)	0.1700	0.0516	0.1147	0.0979
Soleus lipids	^13^C_16_-Phosphatidylcholine (32:0)	0.9825	0.5724	1.0635	0.3921
	^13^C_16_-Triacylglycerol (50:1)	1.1200	0.9231	1.4220	0.8779
	^13^C_16_-Triacylglycerol (52:2)	0.7034	0.5649	1.0165	1.0137
Liver lipids	^13^C_16_-Phosphatidylcholine (32:0)	0.5050	0.2403	0.6007	0.1020
	^13^C_16_-Phosphatidylcholine (34:1)	5.2750	1.9048	6.5153	1.0969
	^13^C_16_-Phosphatidylcholine (34:2)	9.2842	3.7981	11.4464	1.2425
	^13^C_16_-Phosphatidylcholine (34:3)	0.3006	0.1935	0.3611	0.1011
	^13^C_16_-Phosphatidylcholine (38:5)	0.3122	0.1704	0.4211	0.2048
	^13^C_16_-Triacylglycerol (48:0)	0.1192	0.0589	0.2229	0.1387
	^13^C_16_-Triacylglycerol (48:1)	0.4760	0.2129	0.9688	0.5162
	^13^C_16_-Triacylglycerol (50:1)	2.6638	0.9979	3.9606	1.7764
	^13^C_16_-Triacylglycerol (50:2)	4.5403	1.5843	6.7938	2.6669
	^13^C_16_-Triacylglycerol (50:3)	2.3787	0.8710	3.7715	1.2013
	^13^C_16_-Triacylglycerol (52:1)	0.5385	0.2454	0.6731	0.3269
	^13^C_16_-Triacylglycerol (52:2)	7.4767	3.0261	8.7717	3.0478
	^13^C_16_-Triacylglycerol (52:3)	9.8100	3.6473	11.2169	3.1706
	^13^C_16_-Triacylglycerol (52:4)	4.2372	1.8954	4.6790	1.2870
	^13^C_16_-Triacylglycerol (54:6)	2.3202	1.1470	2.0305	0.3243

^*^Significantly different between control and exercised (p = 0.041).
